# Genome-Wide Characterization of the Von Willebrand Factor a Gene Family in Wheat: Highlights Their Functional Roles in Growth and Biotic Stress Response

**DOI:** 10.3390/plants14192965

**Published:** 2025-09-24

**Authors:** Luna Tao, Zheng Yang, Kai Han, Chao Ma, Yueming Ren, Ranran Jia, Huanhuan Li, Qianwen Liu, Yue Zhao, Wenxuan Liu

**Affiliations:** State Key Laboratory of High-Efficiency Production of Wheat-Maize Double Cropping, College of Life Science, Henan Agricultural University, Zhengzhou 450046, China; 15003760433@163.com (L.T.); yz01027@163.com (Z.Y.); hkedu18337253097@163.com (K.H.); 15838209602@163.com (C.M.); r18317591827@163.com (Y.R.); jiarr66@henau.edu.cn (R.J.); lihuanhuanhappy@henau.edu.cn (H.L.); liuwenxuan@henau.edu.cn (W.L.)

**Keywords:** wheat, vWA, phylogenetic tree, expression pattern, biotic stress

## Abstract

Von Willebrand factor A (vWA) genes play important roles in regulating plant growth and development, as well as biotic stresses. However, limited data are available on the contributions of vWA genes to wheat (*Triticum aestivum* L.). In this study, 114 *TavWA* genes were identified in the wheat genome, which were unevenly distributed on 21 chromosomes. According to the phylogenetic analysis, the 114 *TavWAs* were classified into six groups, two of which (G3 and G6) were unique to wheat. Fifty-five homoeologous gene sets among A, B, and D sub-genomes were detected, which play a crucial role in the expansion of the wheat vWA gene family. Analysis of specific spatiotemporal expression patterns showed that more than 50% of *TavWAs* (61 out of 114) exhibited tissue-specific expression. These included 71 *TavWAs* that responded to one or more of the four biotic stress treatments (flg22, chitin, powdery mildew, and stripe rust). Notably, these included *TavWA1-7D*, a recently reported key growth regulator in wheat, suggesting its additional role in biotic stress responses. RT-qPCR analysis indicated that eight genes (*TavWA1-7D*, *TavWA24-2B*, *TavWA36-1D*, *TavWA37-7D*, *TavWA40*, *TavWA47*, *TavWA51*, and *TavWA53*) may play important roles in wheat’s powdery mildew resistance. Collectively, the results of this study provide significant insights for future research on the involvement of vWA genes in the development and stress responses of wheat.

## 1. Introduction

The von Willebrand factor A (vWA) domain is a conserved protein structural module initially identified in the von Willebrand factor (vWF), from which it derives its name [1]. Comprising approximately 200 amino acid residues, the vWA domain exhibits a characteristic α/β Rossmann fold with alternating α-helices and β-strands, and a metal ion-dependent adhesion site critical for ligand binding [2]. This domain has long attracted scientific interest due to its widespread occurrence and diverse functional roles in immune system proteins, extracellular matrix components, as well as in blood coagulation [3]. Notably, vWA domain-containing proteins have been identified across eukaryotes, prokaryotes, and archaea, where they participate not only in cell adhesion and extracellular matrix assembly, but also in ion channel subunits, receptor signaling, and protease regulation [2]. Functionally, the vWA domain mediates protein–protein interactions within multiprotein complexes [4]. In humans, mutations in this domain can lead to protein dysfunction and subsequent disease pathogenesis [5].

In plants, research on vWA domain-containing proteins remains limited, with most studies focused on model organisms such as *Arabidopsis thaliana* and rice. In *Arabidopsis*, at least five genes encoding vWA domain-containing proteins have been reported, namely *AtBON1*, *AtBON2*, *AtBON3*, *MED25*, and *RGLG3*. Three copine proteins—AtBON1, AtBON2, and AtBON3—function redundantly as negative regulators of cell death and defense responses [6]. Further studies demonstrated that *Arabidopsis* BON1 plays a role in growth homeostasis and disease resistance [7]. *AtMED25* has been shown to modulate disease resistance against fungal and bacterial pathogens through jasmonic acid signaling [8], while *RGLG3* plays a coordinated and positive role in fumonisin B1 (FB1)-elicited programmed cell death in plants [9]. In rice, at least seven genes encoding vWA domain-containing proteins have been identified, including *OsBON1*, *OsBON3*, *OsMED25*, *LGD1*, *OsChlD*, *OsGLS1*, and *OsRGLG5*. Similarly to their *Arabidopsis* orthologs, *OsBON1* and *OsBON3* suppress broad-spectrum disease resistance [10]. *OsMED25* is an important regulator of brassinosteroid (BR) signaling [11], *LGD1* pleiotropically regulates vegetative growth and development [12], *OsChlD* affects chlorophyll synthesis and chloroplast development [13], *OsGLS1* is a key regulator of root system architecture [14], and *OsRGLG5* is targeted by the *Magnaporthe oryzae* effector AvrPi9 and positively regulates basal resistance against blast disease [15]. A genome-wide analysis in rice identified 40 vWA-encoding genes, with expression profiling suggesting their involvement in biotic and abiotic stress responses. Among these, *OsvWA9*, *OsvWA18*, *OsvWA36*, and *OsvWA37* emerged as promising candidates for disease resistance [4]. Recently, in maize, loss of *ZmBON1* function resulted in a dwarf phenotype due to impaired brassinosteroid (BR) signaling, highlighting its importance in BR-mediated growth regulation [16]. Collectively, these findings underscore the pivotal role of vWA genes in plant development and biotic stress resistance.

Wheat (*Triticum aestivum* L., 2n = 6x = 42, BBAADD) is one of the world’s most important crops, yet its yield and quality are frequently compromised by biotic stresses such as powdery mildew, Fusarium head blight, and rust diseases. Intriguingly, vWA domain-containing proteins have been implicated in disease resistance in wheat wild relatives. For instance, Lr9, derived from *Aegilops umbellulata*, confers resistance to leaf rust [17], while Pm57, from *Aegilops searsii*, provides protection against powdery mildew [18]. In wheat, the vWA domain has recently been reported in multiple studies. For example, *TavWA1*, a gene carrying this domain, is a critical regulator of wheat growth, influencing photosynthesis, ribosome biogenesis, and nucleosome function [19]. Similarly, *WPA1* acts as a temperature-sensitive regulator of wheat development and grain yield [20], and structural mutations in *RG1* lead to variations in grain size and flag leaf morphology [21]. In addition, *TaAPA2* mutation causes pleiotropic effects on plant architecture [22]. However, these studies all reported the same gene [23], which corresponds to *TavWA1-7D* in this study. To date, only one vWA family gene has been functionally characterized in wheat, and a comprehensive analysis of the vWA gene family in this species remains lacking.

To bridge this knowledge gap, we conducted a genome-wide identification of vWA family genes using the latest high-quality wheat genome assembly. We systematically analyzed their physicochemical properties, phylogenetic relationships, chromosomal distributions, and expression profiles. Additionally, we investigated the transcriptional responses of eight selected *TavWA* genes (*TavWA1-7D*, *TavWA24-2B*, *TavWA36-1D*, *TavWA37-7D*, *TavWA40*, *TavWA47*, *TavWA51*, and *TavWA53*) to powdery mildew infection via Reverse Transcription quantitative Polymerase Chain Reaction (RT-qPCR). This study not only expands our understanding of the vWA gene family in wheat but also provides a foundation for future functional studies of *TavWA* genes in stress adaptation and crop improvement.

## 2. Results

### 2.1. Identification and Physicochemical Property Analysis of TavWA Genes

To identify vWA genes in wheat, we performed a domain-based search against the IWGSC v2.1 reference genome available in the Ensembl Plants database, using the accession IDs PF00092 (Pfam), SM000327 (SMART), and IPR002035 (InterPro) as queries. This initial screening yielded 121 candidate genes putatively encoding proteins containing vWA domain(s). The corresponding protein sequences were further validated for the presence of conserved vWA domains using the SMART and CDD databases. After excluding sequences with incomplete vWA domains, 114 genes were identified as putative vWA family members for further analysis. Detailed information on the 114 *vWA* genes were listed in Table S1. Specifically, the lengths of TavWA proteins varied greatly from 288 amino acids (TavWA18-5A) to 5359 amino acids (TavWA36-1A), and their molecular masses ranged from 30.35 kDa (TavWA18-5A) to 604.78 kDa (TavWA36-1A). The isoelectric points (*p*I) ranged from 4.36 (TavWA46-4D) to 9.79 (TavWA30-2D). The instability indices of TavWA proteins exhibited a broad range, from 29.78 (TavWA52-7A) to 62.70 (TavWA30-2B), with the majority (80 genes) exceeding 40.00, indicating that most members of this family are unstable. The average hydrophilicity values for 113 TavWA proteins (excluding TavWA29-1A) were negative, indicating that these genes were hydrophilic. Subcellular localization predictions also revealed diverse targeting patterns, with 35 TavWA proteins localized to the nucleus, 28 as cytoplasm, 24 as chloroplast, 9 as plasma membrane, 8 as mitochondrion, 4 as peroxisome, 3 as endoplasmic reticulum, 2 as extracellular, and 1 as vacuole (Table S1).

### 2.2. Phylogenetic Relation and Classification of TavWAs

**Figure 1:**
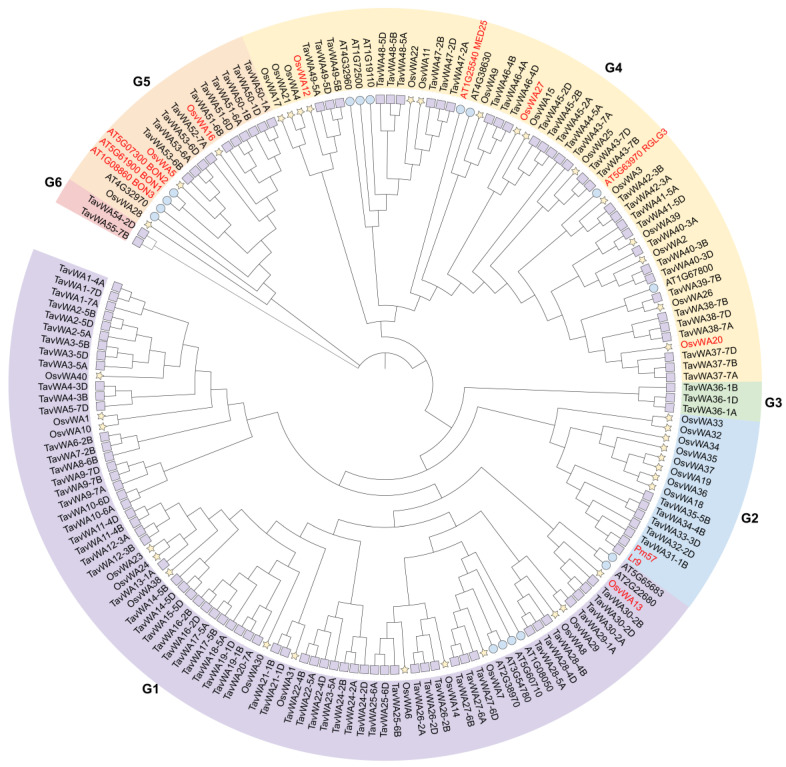
Phylogenetic relationship of TavWA proteins. An unrooted phylogenetic tree was generated using the neighbor-joining method in MEGA 11, incorporating vWA protein sequences from wheat (114), rice (40), *Arabidopsis* (17), along with two wheat relative-derived proteins (Pm57 and Lr9). The wheat vWAs are named according to their positions in the tree. Purple squares represent the TavWA proteins, yellow pentagrams represent the OsvWA proteins, and blue circles represent the AtvWA proteins. G1–G6 represent different groups, and these groups are displayed in different colors. The thirteen characterized vWA proteins are highlighted in red font.

To investigate the evolutionary relationships of TavWA proteins, a phylogenetic tree was reconstructed using 173 vWA protein sequences, comprising 114 from wheat, 17 from *Arabidopsis*, 40 from rice, and two from wheat-related species (Pm57 and Lr9), using the neighbor-joining method (Figure 1). Figure 1 shows that the 114 TavWA genes were classified into six groups (G1–G6). Among them, G1 contained the most TavWA genes (61), followed by G4 (33), G5 (10), G2 (5), G3 (3), and G6 (2). Notably, G3 and G6 exclusively contained vWA proteins from wheat. Genes from different wheat subgenomes but located on adjacent branches were considered different copies of the same *TavWA* gene family member. Based on this, we identified 55 wheat vWA members, designated *TavWA1* to *TavWA55*, with their homoeologous copies distinguished by appending the wheat subgenome symbols A, B, or D.

### 2.3. Chromosomal Distribution and Gene Duplication of TavWA Genes

**Figure 2:**
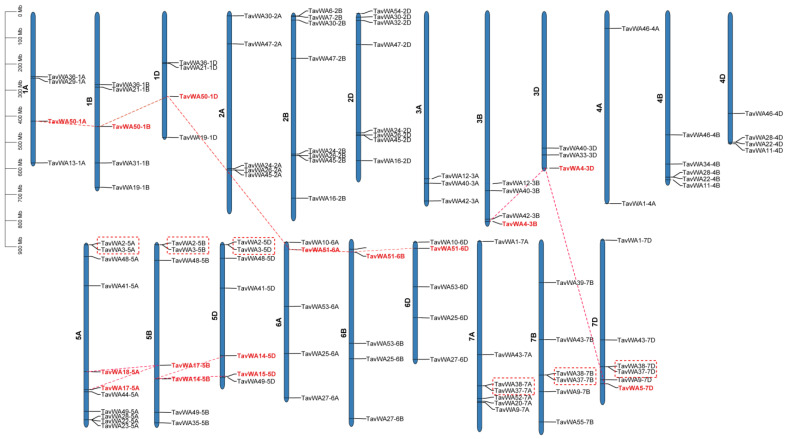
Distribution and duplication of *TavWA* genes on wheat chromosomes. The left scale represents the chromosome length. The scale is in megabases (Mb). The chromosome number is labeled at the left of each chromosome. The tandem duplicated genes were marked with boxes. Segmental duplicated genes are highlighted in red font and marked with red dotted lines.

The *TavWA* genes were found to be unequally distributed on 21 chromosomes of the wheat genome (Figure 2). The number of genes on each chromosome ranged from 2 (chromosome 4A) to 11 (chromosome 5A). Most of the *TavWAs* (65/114) were located on chromosomes groups 2, 5, and 7. Among the 114 *TavWA* genes, there were 37, 40 and 37 members distributed on wheat sub-genomes A, B and D, respectively (Figure 2). In terms of gene duplication, 24 *TavWA* members had the corresponding homoeologous copies on the A, B, and D sub-genomes (Figure 2), indicating that wheat polyploidization was the main reason the expansion of the wheat vWA family. Apart from polyploidization, some genes contain several homologues due to complex gene-duplication events during wheat evolution. Tandem duplication genes were found on chromosome groups 5 and 7 (*TavWA2/TavWA3* and *TavWA37/TavWA38*) based on the analysis criteria. In addition, 8 *TavWA* members (*TavWA4/TavWA5*, *TavWA14/TavWA15*, *TavWA17/TavWA18*, and *TavWA50/TavWA51*) were segmentally duplicated based on their sequence similarity (Figure 2 and Table S2). Furthermore, the *Ka*/*Ks* values for both tandem and segmental duplication gene pairs were less than 1 (0.169565209 to 0.570048974) (Table S3), indicating that purifying selection has been the dominant evolutionary force acting on *TavWA* genes following duplication events.

### 2.4. Expression Analysis of TavWA Genes in Various Wheat Tissues

**Figure 3:**
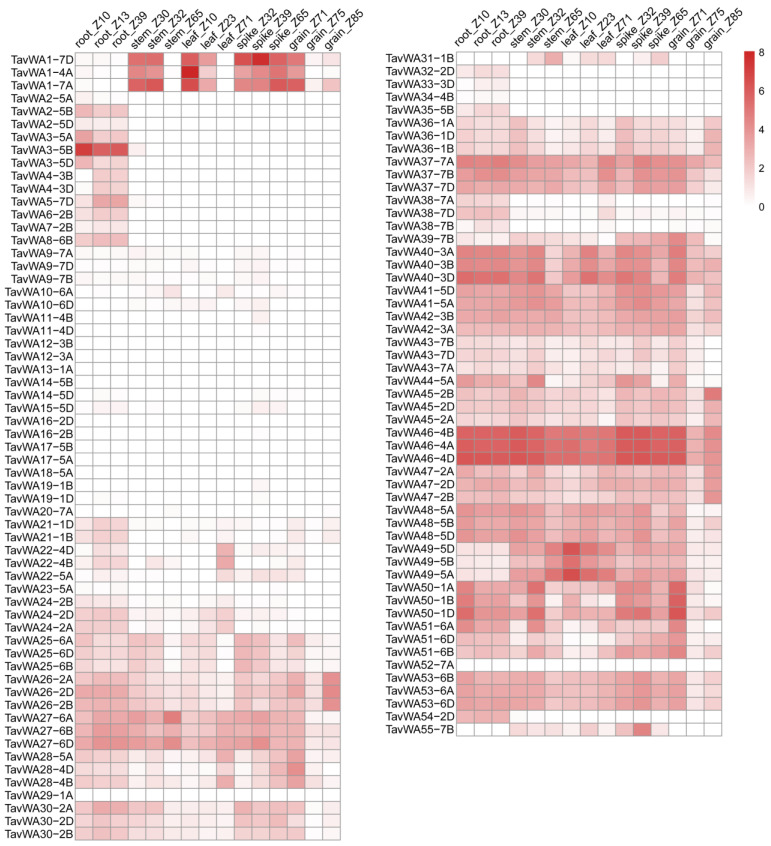
Heat map of *TavWA* genes expression in different tissues and developmental stages. The tissues and developmental stages (Zadoks growth scale) examined are indicated at the top of the heat map. Log2 transformed (FPKM + 1) expression values were used to create the heat map. Colorbar represents the expression abundance of RNA-seq data.

Gene expression patterns are often strongly correlated with gene function. To investigate the expression profiles of *TavWA* genes in wheat roots, stems, leaves, spikes, and grains, we obtained gene expression data from the WheatOmics expression database for various tissues of Chinese Spring across different developmental stages. Of the 114 *TavWA* genes, 28 *TavWAs* (*TavWA26-2A/B/D*, *TavWA36-1A/B/D*, *TavWA37-7A/B/D*, *TavWA40-3A/B/D*, *TavWA41-5A/D*, *TavWA42-3A/B*, *TavWA45-2B/D*, *TavWA46-4A/B/D*, *TavWA47-2A/B/D*, *TavWA50-1D*, and *TavWA53-6A/B/D*) were expressed in all tissues at different developmental stages, whereas 25 *TavWAs* (*TavWA9* to *TavWA20*, *TavWA23-5A*, *TavWA29-1A*, *TavWA34-4B*, and *TavWA52-7A*) showed negligible expression in any tested tissue (Figure 3). The remaining 61 *TavWAs* exhibited tissue-specific expression. In terms of the gene expression patterns among homoeologous genes, most homoeologous gene pairs displayed conserved expression profiles. For example, *TavWA1-4A/7A/7D* were predominantly expressed in young stems, leaves, and spikes and *TavWA2-5A/B/D* expression was strictly root-specific. In contrast, divergent expression patterns were observed for *TavWA50-1A/B/D* and *TavWA51-6A/B/D* (Figure 3), suggesting potential subfunctionalization.

### 2.5. Expression Patterns of TavWA Genes Under Biotic Stresses

**Figure 4:**
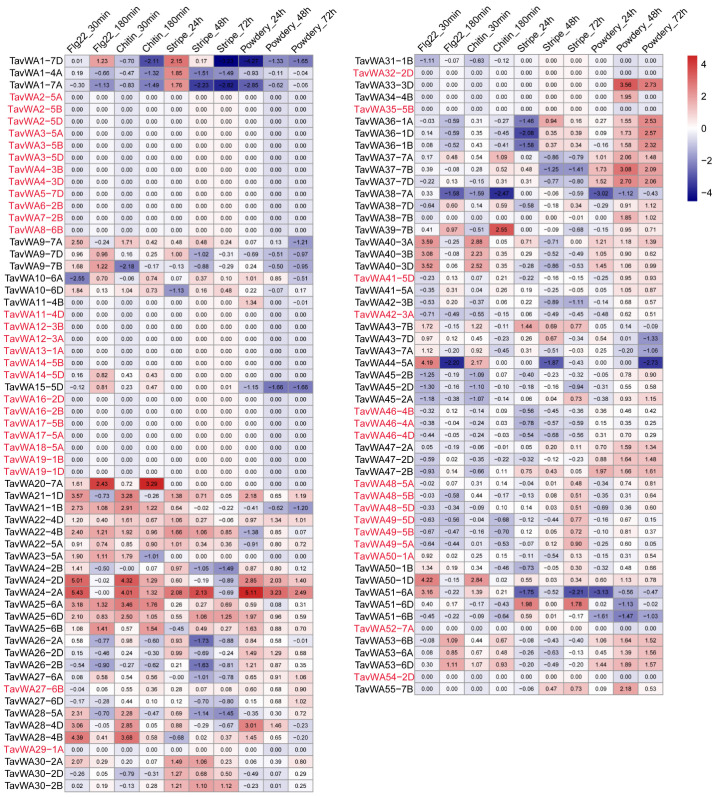
Expression profiles of *TavWA* genes under different biotic stresses. The heatmap displays expression patterns of *TavWA* genes in response to four treatments: flg22 treatment, chitin treatment, stripe rust infection, and powdery mildew infection, with respective time points indicated above the heatmap. Gene expression changes were calculated using RNA-seq data by dividing the FPKM values at each treatment time point by corresponding untreated control values. The color scale represents log2 fold-change values relative to untreated controls. Genes showing no significant expression changes (|log2FoldChange| < 1) under any stress condition are also highlighted in red font.

Previous studies have suggested that the vWA gene family may play a crucial role in plant responses to biotic stresses. To investigate whether *TavWA* genes are involved in biotic stress responses, we analyzed their expression profiles under flg22 (a fragment of bacterial flagellin that elicits immune responses in plants) and chitin treatments, as well as powdery mildew and stripe rust infections. As a result, 43 *TavWA* genes showed no significant expression changes (|log2FoldChange| < 1) across all stress conditions (Figure 4). In contrast, the remaining 71 genes (62.3% of the family) exhibited differential expression in response to at least one stress treatment, with no apparent linear correlation between stress duration and expression magnitude (Figure 4).

Detailed analysis of expression patterns under different stress conditions revealed distinct regulatory responses. Following flg22 treatment, 31 *TavWA* genes exhibited significant upregulation (log2FoldChange ≥ 1) at one or more time points compared to untreated controls, while 8 genes were downregulated (log2FoldChange ≤ −1). Notably, *TavWA44-5A* demonstrated transient induction, showing significant upregulation at 30 min post-treatment but downregulation by 180 min (Figure 4). A comparable expression dynamic was observed during chitin treatment, with 26 genes upregulated and 9 downregulated. The *TavWA23-5A* gene displayed particularly interesting temporal regulation, being upregulated at 30 min but downregulated at 180 min after chitin exposure (Figure 4). Fungal pathogen infections triggered more robust transcriptional responses. Stripe rust infection modulated 32 *TavWA* genes, inducing 15 and repressing 17. Remarkably, 10 genes exhibited time-dependent expression reversals during infection. For instance, *TavWA1-4A/7A/7D* were upregulated at 24 h but downregulated at 48 h and 72 h, while *TavWA36-1A/1B/1D* showed the inverse pattern (Figure 4). Powdery mildew infection affected 50 genes, upregulating 37 and downregulating 13. Among these, 18 genes (including *TavWA24-2A/2B/2D*, *TavWA36-1A/1B/1D*, *TavWA37-7A/7B/7D*, *TavWA40-3A/3B/3D*, *TavWA47-2A/2B/2D*, and *TavWA53-6A/6B/6D*) maintained consistent upregulation across all three examined time points (Figure 4). Notably, *TavWA1-7D*, a wheat growth-related gene, exhibited differential expression patterns under various biotic stress conditions: it was upregulated after 3 h of flg22 treatment or 24 h post stripe rust infection, but downregulated following 3 h of chitin treatment, 72 h post stripe rust infection, and under powdery mildew infection conditions (Figure 4).

**Figure 5:**
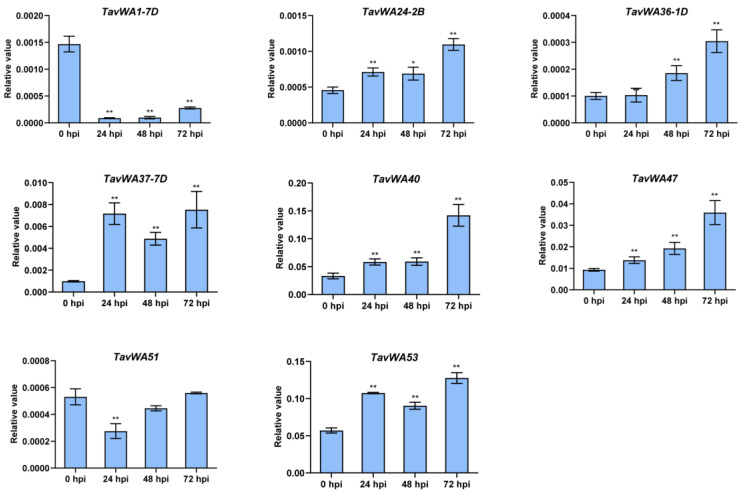
RT-qPCR verification of the expression levels of eight *TavWA* genes under powdery mildew treatment. Plants were maintained in a growth chamber under controlled conditions: 22 °C, 70% relative humidity, and a 16/8-h light/dark cycle. The first leaves of two-leaf seedlings were used to extract total RNA before inoculation (0 h) and 24, 48, and 72 h post-inoculation (hpi) with *Bgt* E26 [24]. Three independent biological replicates were analyzed per time point, with each replicate consisting of pooled tissue from the first leaves of three individual plants. RT-qPCR was performed with three technical replicates per biological replicate. Data were normalized by the *TaActin* gene using the comparative CT method. Values represent means ± SD from three biological replicates. The expression levels of *TavWA40*, *TavWA47*, *TavWA51*, and *TavWA53* represent the combined expression of their three respective homoeologous genes. Asterisks indicate significant differences at each time point compared to the 0 hpi control as determined by two-tailed Student’s *t*-test: *p* < 0.05 (*), *p* < 0.01 (**).

In order to verify the expression of the *TavWA* genes under biotic stress conditions based on the RNA-Seq data, we selected eight *TavWA* genes (*TavWA1-7D*, *TavWA24-2B*, *TavWA36-1D*, *TavWA37-7D*, *TavWA40*, *TavWA47*, *TavWA51*, and *TavWA53*) for RT-qPCR analysis during powdery mildew infection at 0, 24, 48, and 72 h. The results indicated that the expression of these genes detected by RT-qPCR exhibited consistent trends with the RNA-seq results (Figure 5). Collectively, these results demonstrate that *TavWA* genes exhibit diverse temporal expression patterns in response to various biotic stresses, with many genes showing stress-specific regulation and some displaying complex time-dependent expression changes. These findings strongly suggest that the *TavWA* gene family plays important and multifaceted roles in wheat responses to biotic stresses.

## 3. Discussion

Genome-wide analysis of gene families provides a powerful approach for characterizing gene functions and elucidating evolutionary patterns [25]. vWA genes play an important role in the process of plant growth and stress response. However, information on and functions regarding the gene family that comprises the vWA domain in wheat have not been elucidated. In the present study, our identification of 114 *vWA* genes in wheat reveals a notable expansion compared to *Arabidopsis* (17 genes) and rice (40 genes) [4], suggesting potential correlations with genome size and polyploidization events. The wheat vWA family is nearly threefold larger than that of rice, consistent with its hexaploid nature. The molecular weights of different TavWA proteins exhibit large variations (Table S1), indicating potential differences in their structure and composition, which suggests that their functions may also differ. In addition, phylogenetic analysis demonstrated that wheat and *Arabidopsis* vWAs co-cluster in three groups (G1, G4, and G5), indicating their divergence predated the monocot-dicot split. Interestingly, *Arabidopsis* lacks representatives in groups G2, G3, and G6 (Figure 1), implying these clades may have emerged after monocot-dicot divergence. Notably, G3 and G6 appear wheat-specific, likely resulting from lineage-specific evolutionary processes. Subcellular localization predictions revealed diverse organelle targeting (nucleus, cytoplasm, chloroplast, etc.), hinting at functional diversification across cellular compartments.

Gene duplication drives family expansion and functional diversification, enabling environmental adaptation [26]. Wheat (*Triticum aestivum* L., BBAADD) possesses a complex hexaploid genome derived from three related diploid progenitor species [27]. This has led to a highly duplicated genome, in which most hexaploid wheat genes are present in multiple copies across various genomes [28]. In this study, we identified 24 homoeologous *TavWA* gene members, with 37, 40, and 37 *TavWA* genes located on the A, B, and D sub-genomes, respectively (Table S1; Figure 3). These findings suggest that gene loss may have occurred in the wheat *vWA* gene family, leading to the absence of some homoeologous copies. Analysis of gene duplication revealed high sequence similarity between *TavWA1-4A*, *TavWA1-7A*, and *TavWA1-7D*. This observation aligns with prior studies reporting non-homologous translocation events between chromosomes 4A and 7B in hexaploid wheat [29]. It is therefore plausible that *TavWA1-4A* may correspond to the missing *TavWA1-7B* homolog. Segmental and tandem duplications play a crucial role in the expansion of plant gene families [30]. Our duplication analysis revealed that, in addition to polyploidization, tandem and segmental duplications involving *TavWA* genes have contributed to their proliferation in the wheat genome (Figure 2). This indicates that both polyploidization and small-scale duplication events have been important drivers of *TavWA* gene family expansion. To assess evolutionary selection pressures, we calculated the *Ka/Ks* ratios for duplicated gene pairs. All analyzed pairs exhibited *Ka/Ks* ratios < 1 (Table S3), suggesting that these genes have primarily undergone purifying selection, maintaining a high degree of functional conservation. In other words, *TavWA* genes are highly conserved and evolve slowly.

Expression profiling provides critical insights into gene function. To investigate the potential roles of vWA family members in wheat, we systematically examined their expression profiles across different tissues. Our analysis revealed that 89 out of the total *TavWA* genes showed detectable expression in at least one examined tissue (Figure 3). Notably, 61 *TavWA* genes (approximately 68.5% of expressed genes) displayed tissue-specific expression patterns, strongly suggesting their specialized functions in particular developmental processes. It is also noteworthy that most homoeologous gene pairs showed conserved expression patterns across different developmental stages. For example, *TavWA2-5A*, *TavWA2-5B*, and *TavWA2-5D* were predominantly expressed in roots, implying a potential role in root development. This observation also raises the possibility of functional redundancy among these three genes. In contrast, divergent expression profiles were observed for *TavWA50* and *TavWA51*, indicating that these genes may have undergone subfunctionalization or neofunctionalization during wheat evolution. Of particular significance is *TavWA1*, currently the only functionally characterized wheat vWA gene. While its three homoeologs (*TavWA1-7A*, *-4A*, and *-7D*) share similar tissue expression patterns (Figure 3), multiple recent studies have specifically linked only the *TavWA1-7D* copy to wheat growth defects [19,20,21,22,23,31], highlighting the importance of subgenome-specific functional analyses in polyploid species.

The vWA genes are widely involved in plant responses to biotic stress, as evidenced by significant findings. In *Arabidopsis*, the vWA-encoding genes *AtBON1*, *AtBON2*, and *AtBON3* are induced by pathogens [6,32]. These genes exhibit overlapping functions critical for plant viability, acting as negative regulators of the cell death pathway—likely through R-gene-mediated resistance [6]. Further studies revealed that *AtBON1* contributes to growth homeostasis and disease resistance [7]. Additionally, *AtMED25* has been demonstrated to modulate resistance against fungal and bacterial pathogens through jasmonic acid signaling [8]. In rice, *OsBON1* (*OsvWA5*) and *OsBON3* (*OsvWA16*) are induced by multiple pathogens, including *Xoo*, *M. oryzae*, and *R. solani*. These genes negatively regulate disease resistance, with their expression levels and protein localization significantly influencing the balance between immunity and agronomic traits [10]. *OsRGLG5* (*OsvWA20*), which is targeted by the *Magnaporthe oryzae* effector AvrPi9, positively regulates basal resistance to blast disease [15]. A recent genome-wide analysis in rice identified 40 vWA-encoding genes, with expression profiling implicating their roles in biotic stress responses. Among these, *OsvWA9*, *OsvWA18*, *OsvWA36*, and *OsvWA37* emerged as promising candidates for disease resistance [4]. Notably, *OsvWA36* and *OsvWA37* were identified within the panicle blast resistance locus *Pb-bd1* through fine mapping [33]. Furthermore, the wheat resistance genes *Lr9* (against leaf rust) and *Pm57* (against powdery mildew) have also been identified as encoding vWA domain-containing proteins [17,18]. However, the mechanisms underlying their disease resistance remain unclear. It has been hypothesized that proteins such as Lr9 and Pm57, which contain both tandem kinase domain and vWA domain, may employ the vWA domain as a decoy that mimics the target of pathogen effectors, thereby facilitating effector recognition and interception [34].

In this study, four TavWA family members—*TavWA50*, *TavWA51*, *TavWA52*, and *TavWA53*—were clustered into the same phylogenetic group as *Arabidopsis* and rice *BON* genes (Figure 1). Among these, *TavWA51* and *TavWA53* showed significant induction upon pathogen challenge (Figure 4 and Figure 5), implying their potential role in regulating biotic stress responses. Moreover, *TavWA37-7D*, an ortholog of *OsRGLG5* (*OsvWA20*), was strongly upregulated upon powdery mildew infection, suggesting its involvement in wheat biotic stress resistance. Interestingly, *TavWA1-7D*, a previously characterized growth regulator in wheat, also responded to biotic stresses, indicating possible dual functionality. Collectively, these findings imply that *TavWA* genes contribute to plant biotic stress adaptation and merit further investigation.

## 4. Materials and Methods

### 4.1. Experimental Design

This study employed an integrated approach combining in silico bioinformatics analyses with experimental validation to characterize the wheat *vWA* gene family. The core experimental design encompassed three main components: (1) Genome-wide identification and comprehensive bioinformatics characterization of *TavWA* genes (physicochemical properties, phylogeny, chromosomal distribution, duplication, evolution; see Section 4.2); (2) Analysis of *TavWA* gene expression patterns using publicly available RNA-seq data (WheatOmics database) across various developmental stages/tissues and in response to biotic stresses (flg22, chitin, stripe rust, powdery mildew; see Section 4.6); (3) Experimental validation of RNA-seq expression patterns for eight selected *TavWA* genes under powdery mildew infection (*Bgt* isolate E26) at four time points (0, 24, 48, 72 hpi) using RT-qPCR (see Section 4.7 and Section 4.8). Details of specific methods, treatments, and replication for each component are provided in the subsequent sections.

### 4.2. Database Search and Structural Analysis of vWA Domain-Containing Proteins in Wheat

First, all the wheat vWA domain-containing protein sequences were downloaded from the Ensembl Plants database (http://plants.ensembl.org/biomart/martview/) (accessed on 19 March 2025) after a BioMart search using the accession IDs of the vWA domain (PF00092, SM000327, and IPR002035) as queries. Then, the sequences were verified using SMART (http://smart.embl-heidelberg.de/) (accessed on 27 March 2025) and CDD (https://www.ncbi.nlm.nih.gov/Structure/cdd/wrpsb.cgi) (accessed on 27 March 2025) [35] to identify their conserved domains.

### 4.3. Physicochemical Property Analysis and Subcellular Localization of TavWA Proteins

The TavWA protein sequences were submitted to the ExPASy online tool (https://web.expasy.org/protparam/) (accessed on 2 April 2025) to predict their basic physicochemical properties, including the protein molecular weights (MW), isoelectric points (*p*I), instability index, aliphatic Index, and grand average of hydrophobicity. In addition, the online tool Plant-mPLoc (http://www.csbio.sjtu.edu.cn/bioinf/plant-multi) (accessed on 4 April 2025) was used for predicting subcellular localization.

### 4.4. Physical Locations, Multiple Sequence Alignment, and Construction of Phylogenetic Tree

The chromosomal positions of all vWA domain-containing genes were retrieved from the IWGSC RefSeq v2.1 database (https://wheat-urgi.versailles.inra.fr/Seq-Repository/BLAST) (accessed on 2 April 2025) and visualized using MapInspect 1.0 software (https://mapinspect.software.informer.com/download/) (accessed on 11 April 2025). Multiple sequence alignment was performed using ClustalW v2.0 with default parameters [36]. The sequences of *Arabidopsis* and rice vWA-containing proteins [4] were retrieved from the TAIR database (https://www.arabidopsis.org/) (accessed on 2 April 2025) and the Ensembl Plants database (http://plants.ensembl.org/index.html) (accessed on 2 April 2025), respectively. The phylogenetic tree was constructed in MEGA v11 [37] using the Neighbor-Joining (NJ) method (bootstrap = 1000 replicates, other parameters set to default). Final tree visualization was refined using the iTOL web tool (https://itol.embl.de) (accessed on 11 April 2025).

### 4.5. Gene Duplication and Ka/Ks Analysis

To identify duplicated gene pairs within the TavWA family, BLASTP analysis was performed under the following criteria [38]: (1) the alignable region covered over 80% of the longer gene’s length, and (2) the sequence identity within the aligned segment exceeded 80%. The non-synonymous (*Ka*) and synonymous (*Ks*) substitution rates for these duplicated pairs were subsequently computed with KaKs_Calculator 3.0 [39].

### 4.6. Expression Analysis of TavWA Genes During Plant Development and Biotic Stress

Expression profiles of *TavWA* genes were analyzed using publicly available RNA-seq data from the WheatOmics expression database (http://wheatomics.sdau.edu.cn/expression/wheat.html) (accessed on 6 April 2025), which includes datasets generated with appropriate biological replication as per the cited sources [40,41,42]. We utilized the Fragments Per Kilobase of transcript per Million mapped reads (FPKM) normalized values. The tissue-specific analysis included root, stem, leaf, spike, and grain samples, while developmental stage analysis covered the vegetative, reproductive, and mature phases [40], with growth stages classified according to the Zadoks cereal growth scale [43]. For biotic stress responses, we examined four treatment conditions, including treatment with 1 g/l chitin, 500 nM flg22, infection with stripe rust (*Puccinia striiformis* f. sp. *tritici*; *Pst*), and powdery mildew (*Blumeria graminis* f. sp. *tritici*; *Bgt*). The chitin and flg22 treatments comprised three time points (0, 30, 180 min post-treatment) [41], while stripe rust and powdery mildew infections were evaluated at four time points (0, 24, 48, 72 h post-inoculation) [42]. Gene expression patterns were visualized using the R heatmap package (version 4.3.3) with log2(FPKM + 1) transformed values for developmental/tissue data and log2 fold-change values relative to untreated controls for stress data.

### 4.7. Plant Materials and Stress Treatments

Seeds of the wheat cultivar Chinese Spring were surface-sterilized, soaked, germi-nated, and subsequently sown in pots containing a soil mixture. Plants were grown in a controlled environment growth chamber at 22 °C with 70% relative humidity and a 16-h light/8-h dark photoperiod. For the powdery mildew infection experiment, when the first leaves were fully unfolded, seedlings were inoculated using the powdery mildew (*Bgt*) isolate E26 [24]. Inoculation was performed by evenly dusting plants with fresh *Bgt* conidia. The first leaf samples were harvested at 0 h (pre-inoculation control), 24 h, 48 h, and 72 h post-inoculation (hpi). For each time point, leaves were collected from three individual seedlings to form one independent biological replicate. A total of three independent biological replicates were collected per time point. Specifically, for each replicate at each time point, the first leaves from three separate plants were pooled together. All harvested samples were rapidly frozen using liquid nitrogen and stored at −80 °C for subsequent RNA extraction.

### 4.8. RNA Extraction and RT-qPCR Analysis

Total RNA was isolated from the frozen leaf samples using TRIzol reagent (TransGen, Beijing, China) following the manufacturer’s protocol. RNA quality and quantity were assessed using a NanoDrop spectrophotometer (Thermo Scientific, Waltham, MA, USA) and agarose gel electrophoresis (Bio-Rad, Hercules, CA, USA). First-strand cDNA was synthesized from 2 μg of total RNA per sample using a HiScript II 1st Strand cDNA Synthesis Kit (+gDNA wiper) (Vazyme, Nanjing, China). Transcription-quantitative Polymerase Chain Reaction (RT-qPCR) was performed using SYBR Mix (TaKaRa, Dalian, China) on a CFX96 real-time PCR detection system (Bio-Rad, Hercules, CA, USA). The wheat *TaActin* gene was used as the internal reference gene for normalization [44]. Primer sequences for the eight selected *TavWA* genes and *TaActin* are listed in Table S4.

RT-qPCR analysis was conducted on cDNA samples derived from the three independent biological replicates per time point (0, 24, 48, 72 hpi) of the powdery mildew infection experiment. For each biological replicate, each target gene was amplified in three technical replicates. The RT-qPCR conditions were as follows: initial denaturation at 94 °C for 3 min, followed by 40 cycles of 94 °C for 15 s, then 60 °C for 20 s, and 72 °C for 20 s, with a final melt curve analysis step [45]. Relative gene expression levels were calculated using the comparative CT method [46].

### 4.9. Statistical Analyses

Statistical analyses were conducted using GraphPad Prism 8.0 software (GraphPad, San Diego, CA, USA). The differences between the means of groups were determined using Student’s *t*-test. When the *p*-value was below 0.05, the variances were considered significant (*), and highly significant differences were denoted when *p* < 0.01 (**).

## 5. Conclusions

This study provides a comprehensive identification and characterization of the wheat vWA gene family, uncovering its physicochemical properties, phylogenetic relationships, chromosomal distributions, and expression features. A total of 114 *TavWA* genes were identified and classified into six distinct subfamilies, two of which are unique to wheat. Expression profiling revealed that most *TavWA* genes are potentially involved in development and responses to biotic stresses. Many genes exhibited tissue-specific and stress-induced expression patterns, with several showing significant responses to powdery mildew infection. These results establish an important foundation for further functional investigation of *TavWA* genes and support their potential use in molecular breeding for improved biotic stress resistance in wheat.

## Data Availability

Data are contained within the article and Supplementary Materials. Expression data for developmental and biotic stress responses were obtained from public data-bases.

## Supplementary Materials

The following supporting information can be downloaded at https://www.mdpi.com/article/10.3390/plants14192965/s1, Table S1: List of *TavWA* genes identified in wheat; Table S2: Identities between TavWA proteins; Table S3: Analysis of *Ka* and *Ks* ratio of gene replication pairs; Table S4: Primer sequences used in this study.

## References

[1] Colombatti A., Bonaldo P., Doliana R. (1993). Type a modules: Interacting domains found in several non-fibrillar collagens and in other extracellular matrix proteins. Matrix.

[2] Whittaker C.A., Hynes R.O. (2002). Distribution and evolution of von willebrand/integrin a domains: Widely dispersed domains with roles in cell adhesion and elsewhere. Mol. Biol. Cell.

[3] Tuckwell D. (1999). Evolution of von Willebrand factor A (vWA) domains. Biochem. Soc. Trans..

[4] Karkute S.G., Kumar V., Tasleem M., Mishra D.C., Chaturvedi K.K., Rai A., Sevanthi A.M., Gaikwad K., Sharma T.R., Solanke A.U. (2022). Genome-wide analysis of von willebrand factor a gene family in rice for its role in imparting biotic stress resistance with emphasis on rice blast disease. Rice Sci..

[5] Bharati K.P., Prashanth U.R. (2011). Von willebrand disease: An overview. Indian J. Pharm. Sci..

[6] Yang S., Yang H., Grisafi P., Sanchatjate S., Fink G.R., Sun Q., Hua J. (2006). The BON/CPN gene family represses cell death and promotes cell growth in *Arabidopsis*. Plant. J..

[7] Wang Z., Meng P., Zhang X., Ren D., Yang S. (2011). BON1 interacts with the protein kinases BIR1 and BAK1 in modulation of temperature-dependent plant growth and cell death in *Arabidopsis*. Plant J..

[8] Hussein N.K., Sabr L.J., Lobo E., Booth J., Ariens E., Detchanamurthy S., Schenk P.M. (2020). Suppression of *Arabidopsis* mediator subunit-encoding MED18 confers broad resistance against DNA and RNA viruses while MED25 is required for virus defense. Front. Plant Sci..

[9] Zhang X., Wu Q., Cui S., Ren J., Qian W., Yang Y., He S., Chu J., Sun X., Yan C. (2015). Hijacking of the jasmonate pathway by the mycotoxin fumonisin B1 (FB1) to initiate programmed cell death in *Arabidopsis* is modulated by RGLG3 and RGLG4. J. Exp. Bot..

[10] Yin X., Zou B., Hong X., Gao M., Yang W., Zhong X., He Y., Kuai P., Lou Y., Huang J. (2018). Rice copine genes *OsBON1* and *OsBON3* function as suppressors of broad-spectrum disease resistance. Plant Biotechnol. J..

[11] Ren Y., Tian X., Li S., Mei E., He M., Tang J., Xu M., Li X., Wang Z., Li C. (2020). *Oryza sativa* mediator subunit OsMED25 interacts with OsBZR1 to regulate brassinosteroid signaling and plant architecture in rice. J. Integr. Plant Biol..

[12] Thangasamy S., Chen P., Lai M., Chen J., Jauh G. (2012). Rice LGD1 containing RNA binding activity affects growth and development through alternative promoters. Plant J..

[13] Zhang H., Li J., Yoo J.H., Yoo S.C., Cho S.H., Koh H.J., Seo H.S., Paek N.C. (2006). Rice Chlorina-1 and Chlorina-9 encode ChlD and ChlI subunits of Mg-chelatase, a key enzyme for chlorophyll synthesis and chloroplast development. Plant Mol. Biol..

[14] Li Y., Ren M., Wu Y., Wang L., Zhao K., Gao H., Li M., Liu Y., Zhu J., Xu J. (2025). A root system architecture regulator modulates OsPIN2 polar localization in rice. Nat. Commun..

[15] Liu Z., Qiu J., Shen Z., Wang C., Jiang N., Shi H., Kou Y. (2023). The E3 ubiquitin ligase OsRGLG5 targeted by the *Magnaporthe oryzae* effector AvrPi9 confers basal resistance against rice blast. Plant Commun..

[16] Jing T., Wu Y., Yu Y., Li J., Mu X., Xu L., Wang X., Qi G., Tang J., Wang D. (2024). Copine proteins are required for brassinosteroid signaling in maize and *Arabidopsis*. Nat. Commun..

[17] Wang Y., Abrouk M., Gourdoupis S., Koo D.H., Karafiatova M., Molnar I., Holusova K., Dolezel J., Athiyannan N., Cavalet-Giorsa E. (2023). An unusual tandem kinase fusion protein confers leaf rust resistance in wheat. Nat. Genet..

[18] Zhao Y., Dong Z., Miao J., Liu Q., Ma C., Tian X., He J., Bi H., Yao W., Li T. (2024). *Pm57* from *Aegilops searsii* encodes a tandem kinase protein and confers wheat powdery mildew resistance. Nat. Commun..

[19] Chang G., Li Y., Peng L., Shen C., Lu Y., Teng W., Liu Y., Wang Y., Zhu W., Liu C. (2025). *TavWA1* is critical for wheat growth by modulating cell morphology and arrangement. J. Integr. Plant Biol..

[20] Chen Y., Xiao H., Wang Y., Li W., Li L., Dong L., Zhao X., Li M., Lu P., Zhang H. (2024). *WPA1* encodes a vWA domain protein that regulates wheat plant architecture. Crop J..

[21] Zhou C., Xiong H., Jia Y., Guo H., Fu M., Xie Y., Zhao L., Gu J., Li H., Li Y. (2024). Identification of a von Willebrand factor type A protein affecting both grain and flag leaf morphologies in wheat. Sci. China Life Sci..

[22] Bai S., Wang G., Song R., Liu Y., Hua L., Yang J., Zhang L., Ur Rehman S., Hao X., Hou L. (2024). Mutations in wheat *TaAPA2* gene result in pleiotropic effects on plant architecture. Sci. China Life Sci..

[23] Mao L. (2024). One bird, multiple stones: The race to find a gene of dominant negative effect in wheat. Crop J..

[24] Ma C., Tian X., Dong Z., Li H., Chen X., Liu W., Yin G., Ma S., Zhang L., Cao A. (2024). An *Aegilops longissima* NLR protein with integrated CC-BED module mediates resistance to wheat powdery mildew. Nat. Commun..

[25] Wu Y., Feng J., Zhang Q., Wang Y., Guan Y., Wang R., Shi F., Zeng F., Wang Y., Chen M. (2024). Integrative gene duplication and genome-wide analysis as an approach to facilitate wheat reverse genetics: An example in the TaCIPK family. J. Adv. Res..

[26] Magadum S., Banerjee U., Murugan P., Gangapur D., Ravikesavan R. (2013). Gene duplication as a major force in evolution. J. Genet..

[27] Liu J., Yao Y., Xin M., Peng H., Ni Z., Sun Q. (2022). Shaping polyploid wheat for success: Origins, domestication, and the genetic improvement of agronomic traits. J. Integr. Plant Biol..

[28] Bi H., Liu Z., Liu S., Qiao W., Zhang K., Zhao M., Wang D. (2024). Genome-wide analysis of wheat xyloglucan endotransglucosylase/hydrolase (XTH) gene family revealed *TaXTH17* involved in abiotic stress responses. BMC Plant Biol..

[29] Berkman P.J., Skarshewski A., Manoli S., Lorenc M.T., Stiller J., Smits L., Lai K., Campbell E., Kubaláková M., Simková H. (2012). Sequencing wheat chromosome arm 7BS delimits the 7BS/4AL translocation and reveals homoeologous gene conservation. Theor. Appl. Genet..

[30] Cannon S.B., Mitra A., Baumgarten A., Young N.D., May G. (2004). The roles of segmental and tandem gene duplication in the evolution of large gene families in *Arabidopsis thaliana*. BMC Plant Biol..

[31] Zhang L., Zhou H., Fu X., Zhou N., Liu M., Bai S., Zhao X., Cheng R., Li S., Zhang D. (2024). Identification and map-based cloning of an EMS-induced mutation in wheat gene *TaSP1* related to spike architecture. Theor. Appl. Genet..

[32] Liu J., Jambunathan N., McNellis T.W. (2005). Transgenic expression of the von Willebrand A domain of the BONZAI1/ COPINE1 protein triggers a lesion-mimic phenotype in *Arabidopsis*. Planta.

[33] Fang N., Wei X., Shen L., Yu Y., Li M., Yin C., He W., Guan C., Chen H., Zhang H. (2019). Fine mapping of a panicle blast resistance gene *pb-bd1* in japonica landrace bodao and its application in rice breeding. Rice.

[34] Reveguk T., Fatiukha A., Potapenko E., Reveguk I., Sela H., Klymiuk V., Li Y., Pozniak C., Wicker T., Coaker G. (2025). Tandem kinase proteins across the plant kingdom. Nat. Genet..

[35] Wang J., Chitsaz F., Derbyshire M.K., Gonzales N.R., Gwadz M., Lu S., Marchler G.H., Song J.S., Thanki N., Yamashita R.A. (2023). The conserved domain database in 2023. Nucleic Acids. Res..

[36] Larkin M.A., Blackshields G., Brown N.P., Chenna R., McGettigan P.A., McWilliam H., Valentin F., Wallace I.M., Wilm A., Lopez R. (2007). Clustal W and clustal X version 2.0. Bioinformatics.

[37] Tamura K., Stecher G., Kumar S. (2021). MEGA11: Molecular evolutionary genetics analysis version 11. Mol. Biol. Evol..

[38] Zhao Y., Ma R., Xu D., Bi H., Xia Z., Peng H. (2019). Genome-wide identification and analysis of the AP2 transcription factor gene family in wheat (*Triticum aestivum* L.). Front. Plant Sci..

[39] Zhang Z. (2022). KaKs_calculator 3.0: Calculating selective pressure on coding and non-coding sequences. Genom. Proteom. Bioinform..

[40] International Wheat Genome Sequencing Consortium (IWGSC) (2014). A chromosome-based draft sequence of the hexaploid bread wheat (*Triticum aestivum*) genome. Science.

[41] Steuernagel B., Witek K., Krattinger S.G., Ramirez-Gonzalez R.H., Wulff B. (2018). Physical and transcriptional organisation of the bread wheat intracellular immune receptor repertoire. bioRxiv.

[42] Zhang H., Yang Y., Wang C., Liu M., Li H., Fu Y., Wang Y., Nie Y., Liu X., Ji W. (2014). Large-scale transcriptome comparison reveals distinct gene activations in wheat responding to stripe rust and powdery mildew. BMC Genom..

[43] Zadoks J.C., Chang T.T., Konzak C.F. (1974). A decimal code for the growth stages of cereals. Weed Res..

[44] Li H., Men W., Ma C., Liu Q., Dong Z., Tian X., Wang C., Liu C., Gill H.S., Ma P. (2024). Wheat powdery mildew resistance gene *Pm13* encodes a mixed lineage kinase domain-like protein. Nat. Commun..

[45] Zhao Y., He J., Liu M., Miao J., Ma C., Feng Y., Qian J., Li H., Bi H., Liu W. (2024). The SPL transcription factor *TaSPL6* negatively regulates drought stress response in wheat. Plant Physiol. Biochem..

[46] Schmittgen T.D., Livak K.J. (2008). Analyzing real-time PCR data by the comparative C(t) method. Nat. Protoc..

