# Supplementary material for: Genome-Wide Characterization of the Von Willebrand Factor a Gene Family in Wheat: Highlights Their Functional Roles in Growth and Biotic Stress Response
Source: Plants (Basel). 2025 Sep 24;14(19):2965. doi: 10.3390/plants14192965 (PMC12525662; doi:10.3390/plants14192965)
Supplement: Supplementary file 1 [file plants-14-02965-s001.zip › plants-3747340-supplementary.pdf]

Supplementary Tables

Table S1. List of *TavWA* genes identified in wheat.

No.	Gene ID	Generic name	Number of Amino Acid	Molecular Weight	Theoretical pI	Instability Index	Grand Average of Hydropathicity	Predicted subcellular localization
1	TraesCS7D03G0023900	TavWA1-7D	524	55524.57	4.75	32.32	-0.235	cyto
2	TraesCS4A03G1222000	TavWA1-4A	524	55399.43	4.72	30.79	-0.219	cyto
3	TraesCS7A03G0021000	TavWA1-7A	524	55364.33	4.76	32.83	-0.224	chlo
4	TraesCS5A03G0026200	TavWA2-5A	500	53617.18	4.77	34.50	-0.310	cyto
5	TraesCS5B03G0020600	TavWA2-5B	478	51588.94	4.81	40.34	-0.320	chlo
6	TraesCS5D03G0036200	TavWA2-5D	484	52184.53	4.66	37.27	-0.281	cyto
7	TraesCS5A03G0026700	TavWA3-5A	526	56203.20	4.93	41.63	-0.276	cyto
8	TraesCS5B03G0020800	TavWA3-5B	525	56210.19	4.87	37.86	-0.295	cyto
9	TraesCS5D03G0036400	TavWA3-5D	523	55765.79	4.92	36.77	-0.259	cyto
10	TraesCS3B03G1442200	TavWA4-3B	524	56367.93	5.52	34.13	-0.215	cyto
11	TraesCS3D03G1143900	TavWA4-3D	522	56247.91	5.72	32.81	-0.212	cyto
12	TraesCS7D03G1028200	TavWA5-7D	521	55981.98	6.00	31.62	-0.127	cyto
13	TraesCS2B03G0052900	TavWA6-2B	973	109035.35	6.08	48.46	-0.369	pero
14	TraesCS2B03G0056200	TavWA7-2B	599	66861.80	5.14	44.86	-0.346	cyto
15	TraesCS6B03G0135900	TavWA8-6B	504	55847.23	5.72	33.45	-0.161	nucl
16	TraesCS7A03G1036200	TavWA9-7A	725	80690.87	9.42	49.58	-0.359	plas
17	TraesCS7D03G0990700	TavWA9-7D	728	81105.18	9.30	48.25	-0.374	plas
18	TraesCS7B03G0876900	TavWA9-7B	728	81064.19	9.15	48.21	-0.375	plas
19	TraesCS6A03G0012800	TavWA10-6A	707	78017.56	8.65	46.99	-0.118	E.R.
20	TraesCS6D03G0019300	TavWA10-6D	740	81843.89	8.72	49.90	-0.129	E.R.
21	TraesCS4B03G0929200	TavWA11-4B	712	75288.06	6.33	48.82	-0.190	chlo
22	TraesCS4D03G0814200	TavWA11-4D	567	59296.49	5.40	44.68	-0.188	chlo
23	TraesCS3B03G1049100	TavWA12-3B	411	43898.18	5.83	40.21	-0.290	chlo
24	TraesCS3A03G0924900	TavWA12-3A	527	56046.17	5.56	40.18	-0.190	chlo
25	TraesCS1A03G1028200	TavWA13-1A	629	66865.24	6.33	49.70	-0.212	chlo
26	TraesCS5B03G0849600	TavWA14-5B	512	54636.70	6.34	45.79	-0.234	cyto
27	TraesCS5D03G0786600	TavWA14-5D	568	61452.49	8.75	57.48	-0.333	chlo
28	TraesCS5D03G1044400	TavWA15-5D	600	64176.77	7.58	53.39	-0.244	chlo
29	TraesCS2D03G1025200	TavWA16-2D	524	55597.26	7.28	37.00	-0.183	mito
30	TraesCS2B03G1294000	TavWA16-2B	528	56565.21	9.35	39.74	-0.088	chlo
31	TraesCS5B03G0734600	TavWA17-5B	501	53389.44	7.68	43.48	-0.264	cyto
32	TraesCS5A03G0862700	TavWA17-5A	524	55599.10	8.30	42.83	-0.185	chlo
33	TraesCS5A03G0698900	TavWA18-5A	288	30352.32	9.27	37.54	-0.183	chlo
34	TraesCS1B03G1223400	TavWA19-1B	603	63910.02	7.31	41.46	-0.314	chlo
35	TraesCS1D03G0995600	TavWA19-1D	589	62202.89	6.60	41.64	-0.283	nucl
36	TraesCS7A03G1025800	TavWA20-7A	592	62440.15	5.77	45.03	-0.216	nucl
37	TraesCS1D03G0365900	TavWA21-1D	683	72518.43	9.01	55.26	-0.294	nucl

38	TraesCS1B03G0483500	TavWA21-1B	636	67683.92	8.95	56.55	-0.309	nucl
39	TraesCS4D03G0787800	TavWA22-4D	749	79794.50	5.68	57.66	-0.352	nucl
40	TraesCS4B03G0894400	TavWA22-4B	721	77165.69	5.52	57.43	-0.343	nucl
41	TraesCS5A03G1202700	TavWA22-5A	740	79287.18	5.90	56.06	-0.364	nucl
42	TraesCS5A03G1202900	TavWA23-5A	494	53461.45	6.00	40.86	-0.174	chlo
43	TraesCS2B03G0970100	TavWA24-2B	646	68886.47	5.77	50.06	-0.198	nucl
44	TraesCS2D03G0818600	TavWA24-2D	646	68637.19	6.01	47.33	-0.185	nucl
45	TraesCS2A03G0875100	TavWA24-2A	653	69508.29	6.12	46.28	-0.203	nucl
46	TraesCS6A03G0624600	TavWA25-6A	711	77569.94	8.64	52.00	-0.288	nucl
47	TraesCS6D03G0515900	TavWA25-6D	710	77476.81	8.64	54.23	-0.298	nucl
48	TraesCS6B03G0748200	TavWA25-6B	710	77377.74	8.41	55.08	-0.284	nucl
49	TraesCS2A03G0889300	TavWA26-2A	696	75335.61	8.41	60.34	-0.414	mito
50	TraesCS2D03G0832200	TavWA26-2D	692	75080.43	8.73	61.51	-0.410	mito
51	TraesCS2B03G0980100	TavWA26-2B	695	75467.83	8.62	59.89	-0.410	mito
52	TraesCS6A03G0953800	TavWA27-6A	735	79095.40	8.48	44.95	-0.310	nucl
53	TraesCS6B03G1163200	TavWA27-6B	739	79453.77	8.46	46.38	-0.310	nucl
54	TraesCS6D03G0831300	TavWA27-6D	738	79266.51	8.37	45.80	-0.307	nucl
55	TraesCS5A03G1202400	TavWA28-5A	707	76623.40	6.36	51.30	-0.302	mito
56	TraesCS4D03G0787700	TavWA28-4D	707	76571.35	6.48	50.52	-0.293	mito
57	TraesCS4B03G0893000	TavWA28-4B	707	76664.45	6.36	51.86	-0.307	mito
58	TraesCS1A03G0394700	TavWA29-1A	649	69102.80	6.45	48.27	0.018	nucl
59	TraesCS2A03G0084900	TavWA30-2A	760	79917.43	9.75	61.33	-0.372	pero
60	TraesCS2D03G0079200	TavWA30-2D	763	80211.80	9.79	62.01	-0.392	pero
61	TraesCS2B03G0121000	TavWA30-2B	770	80932.54	9.67	62.70	-0.399	pero
62	TraesCS1B03G0954800	TavWA31-1B	783	87843.58	6.23	42.62	-0.320	mito
63	TraesCS2D03G0134600	TavWA32-2D	790	88977.67	5.86	39.71	-0.313	cyto
64	TraesCS3D03G0964900	TavWA33-3D	835	93593.06	6.41	44.03	-0.421	chlo
65	TraesCS4B03G0790000	TavWA34-4B	799	89622.34	5.65	40.02	-0.321	nucl
66	TraesCS5B03G1296000	TavWA35-5B	1585	177023.71	6.18	41.25	-0.308	nucl
67	TraesCS1A03G0385800	TavWA36-1A	5359	604776.26	5.22	42.51	-0.249	plas
68	TraesCS1D03G0360000	TavWA36-1D	5350	604250.74	5.28	42.86	-0.259	plas
69	TraesCS1B03G0467300	TavWA36-1B	5358	604323.65	5.26	42.63	-0.261	plas
70	TraesCS7A03G0925100	TavWA37-7A	457	50507.09	5.82	49.69	-0.543	chlo
71	TraesCS7B03G0771900	TavWA37-7B	456	50462.92	5.77	52.33	-0.567	nucl
72	TraesCS7D03G0888700	TavWA37-7D	457	50482.09	5.82	50.15	-0.537	chlo
73	TraesCS7A03G0925000	TavWA38-7A	441	48591.22	6.46	47.58	-0.507	chlo
74	TraesCS7D03G0888600	TavWA38-7D	454	50252.08	6.71	46.85	-0.511	chlo
75	TraesCS7B03G0771800	TavWA38-7B	443	48986.44	5.65	46.80	-0.523	nucl
76	TraesCS7B03G0365000	TavWA39-7B	399	43716.00	6.11	35.24	-0.434	nucl
77	TraesCS3A03G0966400	TavWA40-3A	436	46841.03	5.40	50.99	-0.456	nucl
78	TraesCS3B03G1110000	TavWA40-3B	467	50277.20	5.59	51.11	-0.412	nucl
79	TraesCS3D03G0899800	TavWA40-3D	466	50229.11	5.66	52.51	-0.422	nucl
80	TraesCS5D03G0302100	TavWA41-5D	537	58242.91	5.57	57.70	-0.212	extr
81	TraesCS5A03G0267900	TavWA41-5A	559	60736.94	6.24	58.67	-0.231	extr

82	TraesCS3B03G1415800	TavWA42-3B	379	41692.87	5.86	32.85	-0.318	nucl
83	TraesCS3A03G1184000	TavWA42-3A	382	41916.18	5.99	33.76	-0.288	nucl
84	TraesCS7B03G0590800	TavWA43-7B	420	45562.34	5.95	48.46	-0.333	vacu
85	TraesCS7D03G0730500	TavWA43-7D	406	43988.66	6.47	43.70	-0.284	chlo
86	TraesCS7A03G0761300	TavWA43-7A	401	43551.29	6.52	49.32	-0.285	cyto
87	TraesCS5A03G0888300	TavWA44-5A	316	34718.12	5.13	54.50	-0.214	chlo
88	TraesCS2B03G0981700	TavWA45-2B	430	47412.73	5.91	55.91	-0.330	nucl
89	TraesCS2D03G0832900	TavWA45-2D	430	47368.71	5.91	56.11	-0.322	nucl
90	TraesCS2A03G0889600	TavWA45-2A	430	47248.63	5.83	53.39	-0.290	nucl
91	TraesCS4B03G0621900	TavWA46-4B	404	42641.57	4.42	38.10	-0.297	cyto
92	TraesCS4A03G0143100	TavWA46-4A	401	42275.13	4.36	38.77	-0.278	cyto
93	TraesCS4D03G0551400	TavWA46-4D	401	42247.08	4.36	37.68	-0.285	cyto
94	TraesCS2A03G0354300	TavWA47-2A	741	81355.43	5.51	36.10	-0.086	plas
95	TraesCS2D03G0370200	TavWA47-2D	741	81327.41	5.64	34.28	-0.085	plas
96	TraesCS2B03G0473600	TavWA47-2B	741	81389.58	5.71	35.32	-0.087	plas
97	TraesCS5A03G0137900	TavWA48-5A	755	83102.16	5.65	40.60	-0.136	cyto
98	TraesCS5B03G0154000	TavWA48-5B	754	82937.03	5.78	41.37	-0.111	cyto
99	TraesCS5D03G0160800	TavWA48-5D	755	82979.01	5.62	39.97	-0.131	cyto
100	TraesCS5D03G1056600	TavWA49-5D	815	88516.89	6.18	43.05	-0.344	E.R.
101	TraesCS5B03G1166800	TavWA49-5B	761	82552.07	5.43	42.94	-0.303	chlo
102	TraesCS5A03G1099300	TavWA49-5A	761	82610.17	5.43	43.89	-0.305	chlo
103	TraesCS1A03G0613700	TavWA50-1A	590	64936.66	5.68	33.89	-0.185	cyto
104	TraesCS1B03G0700700	TavWA50-1B	590	65012.74	5.73	34.61	-0.186	cyto
105	TraesCS1D03G0577800	TavWA50-1D	590	64798.46	5.76	35.02	-0.193	cyto
106	TraesCS6A03G0131200	TavWA51-6A	633	69248.63	5.78	40.44	-0.173	cyto
107	TraesCS6D03G0119200	TavWA51-6D	590	64853.49	5.52	38.98	-0.211	cyto
108	TraesCS6B03G0174000	TavWA51-6B	590	64961.70	5.52	38.21	-0.189	nucl
109	TraesCS7A03G1002100	TavWA52-7A	588	64970.55	8.63	29.78	-0.122	cyto
110	TraesCS6B03G0678800	TavWA53-6B	577	63222.64	5.23	36.66	-0.149	nucl
111	TraesCS6A03G0483400	TavWA53-6A	574	63103.65	5.23	34.63	-0.135	nucl
112	TraesCS6D03G0404200	TavWA53-6D	574	62960.44	5.18	34.89	-0.139	nucl
113	TraesCS2D03G0004200	TavWA54-2D	586	64551.27	6.01	47.90	-0.157	cyto
114	TraesCS7B03G1155400	TavWA55-7B	1494	166680.22	6.04	41.93	-0.341	chlo

Note: chlo, chloroplast; cyto, cytoplasm; E.R., endoplasmic reticulum; extr, extracellular; mito, mitochondrion; nucl, nucleus; pero, peroxisome; plas, plasma membrane; vacu, vacuole.

Table S2. Identities between TavWA proteins. The identity was estimated when the query coverage was >80%, by nucleotide-nucleotide Basic Local Alignment Search Tool in the National Center for Biotechnology Information databases.

% of coverage / % of identity	TavWA2-5A	TavWA2-5B	TavWA2-5D	TavWA3-5A	TavWA3-5B	TavWA3-5D	TavWA4-3B	TavWA4-3D	TavWA5-7D	TavWA14-5B	TavWA14-5D	TavWA15-5D	TavWA17-5A	TavWA17-5B	TavWA18-5A	TavWA37-7A	TavWA37-7B	TavWA37-7D	TavWA38-7A	TavWA38-7B	TavWA38-7D	TavWA50-1A	TavWA50-1B	TavWA50-1D	TavWA51-6A	TavWA51-6B	TavWA51-6D
**TavWA2-5A**	100	99	100	100	100	100																					
**TavWA2-5B**	85	100	100	100	100	100																					
**TavWA2-5D**	86.3	91.7	100	100	100	100																					
**TavWA3-5A**	82.4	81.9	83.7	100	100	99																					
**TavWA3-5B**	85	84.1	85.7	94.3	100	99																					
**TavWA3-5D**	84.4	83.3	85.1	95.6	97.7	100																					
**TavWA4-3B**							100	99	100																		
**TavWA4-3D**							97.1	100	100																		
**TavWA5-7D**							82.1	82.4	100																		
**TavWA14-5B**										100	100	100															
**TavWA14-5D**										86.5	100	96															
**TavWA15-5D**										86.6	98	100															
**TavWA17-5A**													100	100	81												
**TavWA17-5B**													83.9	100	86												
**TavWA18-5A**													86.3	80.8	100												
**TavWA37-7A**																100	98	99	84	87	88						
**TavWA37-7B**																97.8	100	98	86	88	90						
**TavWA37-7D**																99.1	97.6	100	84	87	88						
**TavWA38-7A**																84.8	86.2	84.6	100	88	93						
**TavWA38-7B**																87	88.4	86.8	88	100	91						
**TavWA38-7D**																88.1	89.7	87.9	93.7	91	100						
**TavWA50-1A**																						100	99	97	91	93	92
**TavWA50-1B**																						98.8	100	97	91	92	91
**TavWA50-1D**																						97.4	97.3	100	90	92	91
**TavWA51-6A**																						91.7	91.5	90.7	100	97	97
**TavWA51-6B**																						93.1	92.7	91.7	97.5	100	97
**TavWA51-6D**																						92.2	91.9	91	97.1	97.3	100

Table S3. Analysis of *Ka* and *Ks* ratio of gene replication pairs. The *Ka* (non-synonymous substitution) and *Ks* (synonymous substitution) substitution rates was calculated using KaKs_Calculator 3.0.

Seq_1	Seq_2	*Ka*	*Ks*	*Ka/Ks*
TavWA50-1A	TavWA51-6B	0.029809539	0.133251357	0.223709086
TavWA50-1B	TavWA51-6B	0.032242639	0.140584866	0.229346441
TavWA50-1D	TavWA51-6B	0.040106296	0.16601851	0.241577254
TavWA2-5A	TavWA3-5A	0.083369947	0.247377596	0.337014945
TavWA2-5B	TavWA3-5B	0.079734085	0.18658835	0.427326172
TavWA2-5D	TavWA3-5D	0.073786215	0.173520536	0.425230447
TavWA4-3B	TavWA5-7D	0.104333542	0.213212302	0.489341099
TavWA4-3D	TavWA5-7D	0.104309707	0.216823992	0.481080097
TavWA37-7A	TavWA38-7A	0.053243156	0.235711719	0.225882513
TavWA37-7B	TavWA38-7B	0.044531501	0.262621683	0.169565209
TavWA37-7D	TavWA38-7D	0.050472902	0.223687941	0.2256398
TavWA17-5B	TavWA18-5A	0.166605029	0.324766211	0.512999886
TavWA17-5A	TavWA18-5A	0.182780127	0.307759028	0.59390663
TavWA14-5B	TavWA15-5D	0.060676985	0.106441706	0.570048974
TavWA14-5D	TavWA15-5D	0.023952219	0.046189333	0.518566032

Table S4. Primer sequences used in this study.

Primer name	Sequence (5´-3´)
TavWA1-7D-qF	TGGACCTGGTGGCGGTGCTG
TavWA1-7D-qR	GCTGCGTGACGGAGCGGAGT
TavWA24-2B-qF	GCACTACTCGGCCGTCGCT
TavWA24-2B-qR	CTCCTGACATCGAGCACCGTA
TavWA36-1D-qF	GATACACAGGAAATATGGCCAAGAG
TavWA36-1D-qR	GCAGTTCGCATTCGTTGAAGATAT
TavWA37-7D-qF	CTCTTCGGCGGGTAACAACA
TavWA37-7D-qR	AATAAGATTTGAAGATTCGAGGCCT
TavWA40-qF	GTCGTCGTTCTATGAAGGCTC
TavWA40-qR	AGGCAGATAGGGCAAACCTG
TavWA47-qF	TGGAGGGTCCGAGTCGTAGT
TavWA47-qR	TCCGACCATTTGGGCTAGTC
TavWA51-qF	GGATGGAGTTATCACCGACG
TavWA51-qR	CTGCCTGTCGAGCTTTGAAG
TavWA53-qF	TGGCGGCAAGTCTTTAGAAG
TavWA53-qR	AGATAAGCAGCAACCAGCATG
TaActin-F	TATGTTCCCGGTATTGCTG
TaActin-R	AGACCCAGACAACTCGCAAC
